# Species-Specific Patterns of Gut Metabolic Modules in Dutch Individuals with Different Dietary Habits

**DOI:** 10.1128/msphere.00512-22

**Published:** 2022-11-17

**Authors:** Sudarshan A. Shetty, Paul B. Stege, Joost Hordijk, Esther Gijsbers, Cindy M. Dierikx, Engeline van Duijkeren, Eelco Franz, Rob J. L. Willems, Fernanda L. Paganelli, Susana Fuentes

**Affiliations:** a Centre for Infectious Disease Control, National Institute for Public Health and the Environment (RIVM), Bilthoven, The Netherlands; b Department of Medical Microbiology and Infection prevention, Virology and Immunology research Group, University Medical Center Groningen, Groningen, The Netherlands; c Department of Medical Microbiology, UMC Utrecht, Utrecht, The Netherlands; University of Michigan-Ann Arbor

**Keywords:** diet, microbiome, omnivore, vegan, diet

## Abstract

Diet is an important determinant of the human gut microbiome. Here, we analyzed fecal metagenomes of Dutch adults following omnivorous, pescatarian, vegan, and vegetarian diets. We compared the taxonomic composition of individuals from our study with publicly available gut metagenomes from westernized and non-westernized societies. We observed that, despite long-term transition to diets rich in plant fibers (vegan or vegetarian), the microbiomes of these were typical of westernized populations, and similar in composition to omnivores. Although there were no major differences in metabolic modules, we identified differences in the species that contributed to particular functions, such as carbohydrate degradation and short-chain fatty acid metabolism. Overall, this study shows functional redundancy of the microbiomes among westernized populations, which is independent of long-term individual dietary habits.

**IMPORTANCE** Diet is an important modulator of the human gut microbiome, which is susceptible to increased consumption of plant fibers in vegan or vegetarian lifestyles. To investigate this, we compared the gut microbiome of Dutch adults following omnivorous, pescatarian, vegan and vegetarian diets. We did not observe major differences in the gut microbiome composition and function between individuals with different dietary habits. However, we observed differences in the species that contribute to the core functions of the gut microbiome. Our study thus emphasizes the need to better understand the species-specific functional changes associated with dietary habits in the human gut microbiome.

## OBSERVATION

In westernized societies traditionally known to have an omnivorous diet, some individuals have transitioned toward vegetarian and vegan diets. These diets are enriched in microbiota-accessible carbohydrates (MACs) that are not digested and absorbed by the host but support a complex microbiome with supposedly beneficial effect on host health (1). MACs like resistant starch, inulin, xylan, pectin and similar complex substrates favor the growth of specialized taxa like Ruminococcus bromii, Roseburia intestinalis, and Faecalibacterium prausnitzii (2). High-fiber diets rich in MACs are reported to have beneficial effects on host physiology via enhanced butyrate production and promoting higher bacterial diversity (3). Short-term (4-day) dietary interventions have reported reversible changes in the microbiome, indicating rapid adaptation of the microbiota to the changing diet (3). Individuals consuming high-fiber diets for longer periods are shown to have a *Prevotella* enriched microbiome, which is stable at least up to 10 days, indicating that long-term dietary habits can select for specific taxa that form a stable community (4). However, in our recent study comparing gut microbiome of individuals with different long-term dietary habits (≥6 months), we did not observe major differences in microbiome diversity and resistome (5). As high functional redundancy is a hallmark of the gut microbiome, where multiple species can carry out similar functional roles (6, 7), linking abundances of specific bacterial taxa with their contribution to functional potential may provide a better understanding of species-specific associations with dietary habits.

Here, we investigated whether different long-term dietary habits (≥6 months) result in a gut microbiome composition that deviates from that observed in westernized populations and identify differences in species-specific contributions to key metabolic pathways.

**FIG 1:**
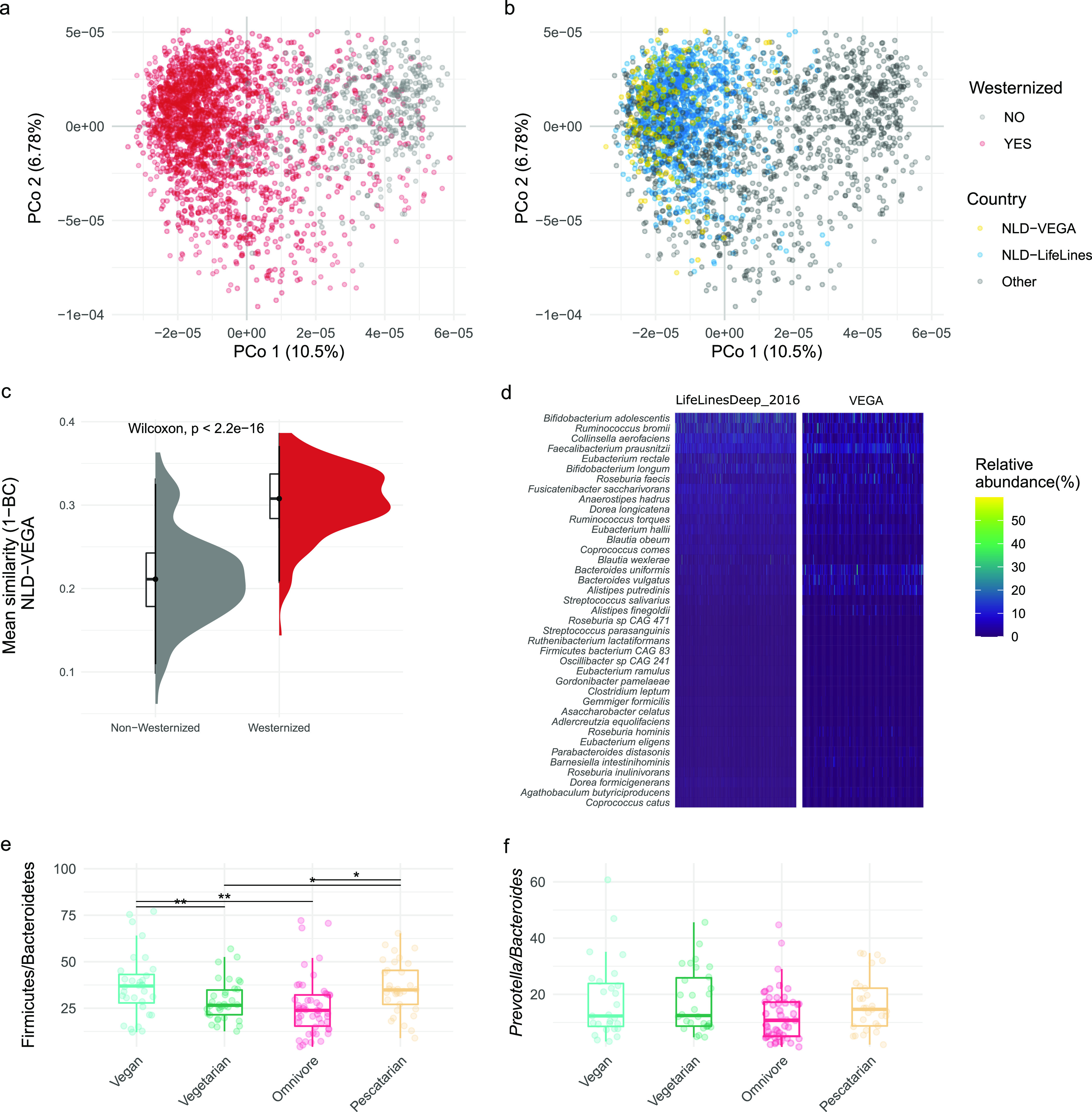
Global and local comparison of gut microbiome of NLD-VEGA. Ordination plot depicting gut microbiome variation between (a) westernized and non-westernized populations and (b) highlighting the overlap between the two Dutch (NLD) populations, i.e., the Lifelines cohort (NLD-Lifelines; *n* = 1039) and our study population (NLD-VEGA; *n* = 149). (c) Similarity (depicted as (1-Bray-Curtis dissimilarity index) of the gut microbiome structure and composition of NLD-VEGA compared to other westernized (N = 1940; from NLD-Lifelines, Denmark, DNK; Ireland, IRL; Great Britain, GBR; Italy, ITA; El Salvador, SLV; USA, USA; China, CHN) and non-westernized populations (N = 429; from Mongolia, MNG; Indonesia, IDN; Tanzania, TZA; Peru, PER; Madagascar, MDG; India, IND; Cameroon, CMR). (d) Abundance of common core species in NLD-VEGA and NLD-Lifelines. Ratios of (e) Firmicutes/Bacteroidetes and f) *Prevotella*/*Bacteroides* in NLD-VEGA participants with different dietary patterns (two-sided Wilcoxon test adjusted by the Benjamini & Hochberg method).

The participants in our study (NLD-VEGA) were classified into vegetarian, vegan or pescatarian if they followed these diets for at least 6 months, and omnivores if they eat meat at least three time per week (5, 8). Details about the NLD-VEGA study population are described in Table S1, and metagenomic data analysis is provided in the supplementary information (Text S1). Information on public data used in this study are provided in Table S2. Taxonomic composition and functional annotations were done using the biobakery tools (9). The fecal microbiomes of participants in the NLD-VEGA study were similar to westernized populations (Wilcox test, *P < *2.2e − 16) and showed high similarity with individuals from another independent Dutch study, Netherlands LifeLines cohort (NLD-LifeLines; *n* = 1039) (Fig. 1a, b and c) (10). The NLD-VEGA and NLD-LifeLines cohorts shared 39 (61%) of the core microbes (min. relative abundance 0.0001% and 75% prevalence) (Fig. S1). This common core (NLD-VEGA + NLD-LifeLines) consists of species involved in polysaccharide degradation and short-chain fatty acid production (e.g., Ruminococcus bromii, Eubacterium rectale, Faecalibacterium prausnitzii, Eubacterium hallii, Coprococcus catus, Anaerostipes hadrus) (Fig. 1d) (11–13).

10.1128/msphere.00512-22.1 FIG S1 Shared core microbes between NLD-LifelinesDeep and NLD-VEGA cohorts. The core microbiota for each of the studies were chosen using 100 bootstraps and present at min. relative abundance of 0.0001% and 75% prevalence. Download FIG S1, PDF file, 0.01 MB. Copyright © 2022 Shetty et al. 2022 Shetty et al. https://creativecommons.org/licenses/by/4.0/ This content is distributed under the terms of the Creative Commons Attribution 4.0 International license.

10.1128/msphere.00512-22.4 TABLE S1 Overview of participants from the NLD-VEGA-study. Download Table S1, DOCX file, 0.02 MB. Copyright © 2022 Shetty et al. 2022 Shetty et al. https://creativecommons.org/licenses/by/4.0/ This content is distributed under the terms of the Creative Commons Attribution 4.0 International license.

10.1128/msphere.00512-22.6 TEXT S1 The supplemental text includes information on data used in this study, bioinformatics processing, and data analysis. Download Text S1, DOCX file, 0.03 MB. Copyright © 2022 Shetty et al. 2022 Shetty et al. https://creativecommons.org/licenses/by/4.0/ This content is distributed under the terms of the Creative Commons Attribution 4.0 International license.

10.1128/msphere.00512-22.5 TABLE S2 Overview of stool metagenome data from the curatedMetagenomicData resource used in present study. We selected samples from each of these studies that were identified as “healthy” and categorized as “adults” in age category. Download Table S2, DOCX file, 0.02 MB. Copyright © 2022 Shetty et al. 2022 Shetty et al. https://creativecommons.org/licenses/by/4.0/ This content is distributed under the terms of the Creative Commons Attribution 4.0 International license.

We investigated the effect of long-term dietary habits (i.e., omnivore, vegetarian, vegan and pescatarian) on so-called VANISH (volatile and/or associated negatively with industrialized societies of humans) taxa in our cohort (14). VANISH taxa, i.e., Succinovibrionaceae, Paraprevotellaceae, Prevotellaceae, and Spirochaetaceae, are considered to be lost with industrialization, and therefore more prevalent in non-westernized microbiomes. As previously observed in westernized diets (14, 15), the NLD-VEGA population showed loss of certain VANISH taxa and overall low abundance of Prevotellaceae (Fig. S2a), with no significant differences between the diet groups. The BloSSUM taxa (bloom or selected in societies of urbanization/modernization) were observed in all diet groups in the NLD-VEGA (Fig. S2b). Furthermore, western-style diet is often associated with a higher Bacteroidetes, while traditional high-fiber diet rich in vegetables and fish is associated with a high Firmicutes abundance (1, 16). Here, we observed a significantly higher Firmicutes/Bacteroidetes ratio in the pescatarian and vegan group compared to the omnivore and vegetarian group (Fig. 1e). While diets rich in plant-based fibers were also reported to be associated with a higher *Prevotella*/*Bacteroides* (P/B) ratio (17), we did not find these associations in our data set (Fig. 1f). These results highlight the potential non-universality of previously reported associations of higher P/B ratio with a plant-based diet (18). In addition to diet, possibly other factors (e.g., environmental exposures) play a significant role in determining the P/B ratio in non-westernized individuals. Therefore, increasing dietary fibers or becoming vegetarian/vegan alone might not be enough to increase the P/B ratio in westernized individuals.

10.1128/msphere.00512-22.2 FIG S2 VANISH and BloSSUM taxa in the NLD-VEGA. VANISH taxa are volatile and/or associated negatively with industrialized societies of humans. BloSSUM taxa are bloom or selected in societies of urbanization/modernization. Download FIG S2, PDF file, 0.01 MB. Copyright © 2022 Shetty et al. 2022 Shetty et al. https://creativecommons.org/licenses/by/4.0/ This content is distributed under the terms of the Creative Commons Attribution 4.0 International license.

**FIG 2:**
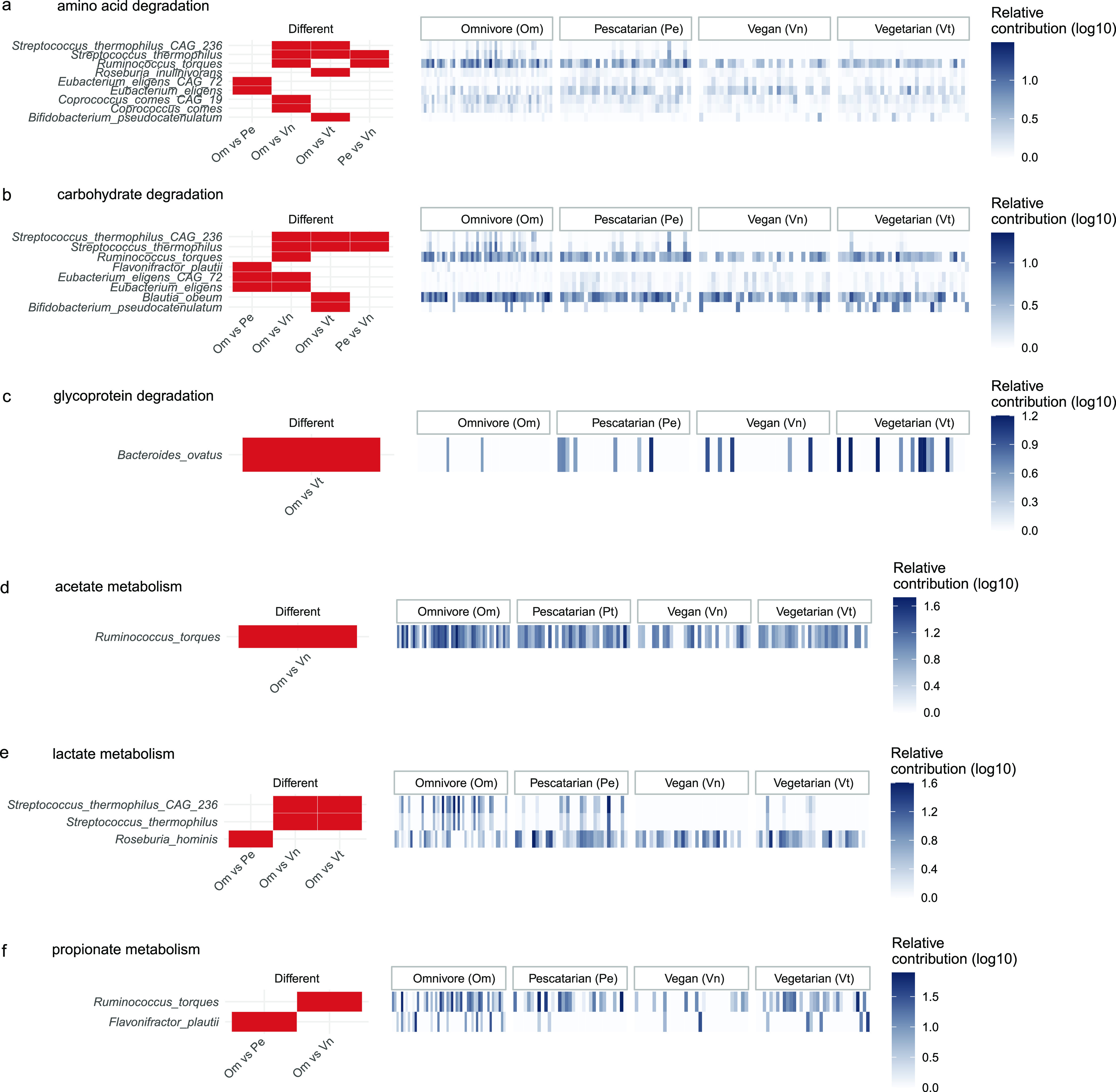
Diet based comparison of gut metabolic modules and contribution by taxa. Species significantly different between diet groups and their relative contribution to gut metabolic modules for degradation (a, b, and c). Species significantly different between diet groups and their relative contribution to gut metabolic modules for short-chain fatty acid metabolism (d, e, and f). Significance in differences were calculated using Kruskal-Wallis test followed by Dunn's test for pairwise comparisons and adjusted for multiple comparisons with the Benjamini & Hochberg method. The differences were considered significant when adjusted *P*-value was less than < 0.05.

Metabolic potential of the gut microbiome is key for maintenance of host health. Here, we investigated the functional repertoire in gut metagenomes and species-specific contributions to key metabolic processes, and its relation to the different long-term dietary habits. We used a curated version of the previously described Gut Metabolic Modules (GMMs) to group KEGG orthologues (KOs) into metabolic modules (see supplementary data) (18). To these GMMs, we added modules related to amino acid metabolism and refined GMMs based on a synthetic microbiome used in a previous study (19). Except for lipid degradation, abundances of amino acid, carbohydrate and glycoprotein degradation modules were not significantly different between diet groups (Fig. S3). However, species-specific differences in abundances of the bacteria involved in the different processes were observed (Fig. 2). We observed nine species that differed in their contribution to amino acid degradation module (Fig. 1a, Kruskal-Wallis test, adj. *P < *0.05, followed by Dunn’s Test adj. *P < *0.05). For carbohydrate degradation module, eight species differed in their contribution between groups (Fig. 1b). No species were observed to differ in relative contribution to amino acid and carbohydrate degradation modules between vegans and vegetarians. For glycoprotein and lipid degradation, Bacteroides ovatus was observed to be significantly different between omnivore and vegetarian (Fig. 1c). Notably, no statistically significant species-specific differences in contribution to lipid metabolism was observed in our study.

10.1128/msphere.00512-22.3 FIG S3 Comparison of gut metabolic module abundances between diet groups in NLD-VEGA. Significance in differences were calculated using two-sided Wilcoxon test adjusted by the Benjamini & Hochberg method. The median counts RPKs stand for reads per kilobase. Download FIG S3, PDF file, 0.3 MB. Copyright © 2022 Shetty et al. 2022 Shetty et al. https://creativecommons.org/licenses/by/4.0/ This content is distributed under the terms of the Creative Commons Attribution 4.0 International license.

Except for butyrate metabolism, the GMMs for acetate, propionate and lactate metabolisms did not differ between diet groups (Fig. S3). Similar with higher level GMMs, species-specific differences in contribution to these modules were observed. Ruminococcus torques had a significantly higher contribution in omnivores compared to vegan group. Three species showed differences in contribution to lactate metabolism, Streptococcus thermophilus and S. thermophilus CAG236 in omnivores compared to vegan and vegetarian groups. Roseburia hominis was higher contributor in pescatarians compared to omnivores. Ruminococcus torques was higher contributor to propionate metabolism module in omnivores. Although *R. torques* is not yet reported to produce propionate, it does have key genes for producing propionate via the propanediol pathway (20). *R. torques* is known to produce lactate, an important precursor for propionate production via the acrylate pathway.

*R. torques* is reported to be associated with proinflammation (21). Flavonifractor plautii contributed higher to propionate metabolism module in omnivores compared to pescatarians. This bacterium is known to utilize simple sugars and amino acids to produce butyrate and was recently shown to be associated with young-onset colorectal cancer (22, 23). No statistically significant species-specific differences in contribution to butyrate metabolism between the four diet group was observed in our study.

Overall, we see that despite showing modest differences in abundances of key GMMs, species-specific differences in contribution to key metabolic pathways were observed between the diet groups. This further highlights the widely accepted functional redundancy of the gut microbiome. Future research investigating species specific activity using a combination of multi-omics and *in vitro*/*in vivo* investigations can help to improve our understanding of diet mediated effects on human health.

### Data availability.

The raw sequencing data are available at EMBL-ENA under accession number PRJEB45944. The codes to reproduce the analysis are available from GitHub (https://github.com/RIVM-IIV-Microbiome/VEGA-2021).
