# Supplementary figures and images for: Species-Specific Patterns of Gut Metabolic Modules in Dutch Individuals with Different Dietary Habits
Source: mSphere. 2022 Nov 17;7(6):e00512-22. doi: 10.1128/msphere.00512-22 (PMC9769759; doi:10.1128/msphere.00512-22)

LifeLines Core Microbiome VEGA Core Microbiome

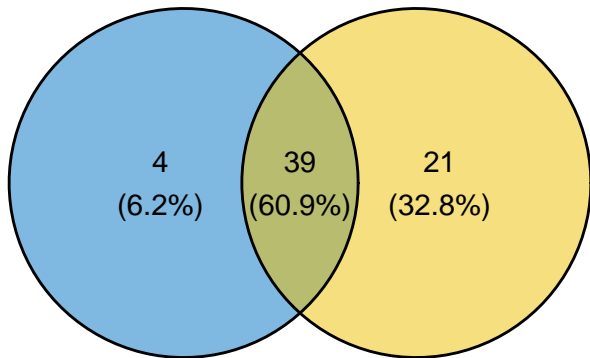

Supplement: FIG S1 [file msphere.00512-22-s0001.pdf]

a

## VANISH taxa in NLD-VEGA

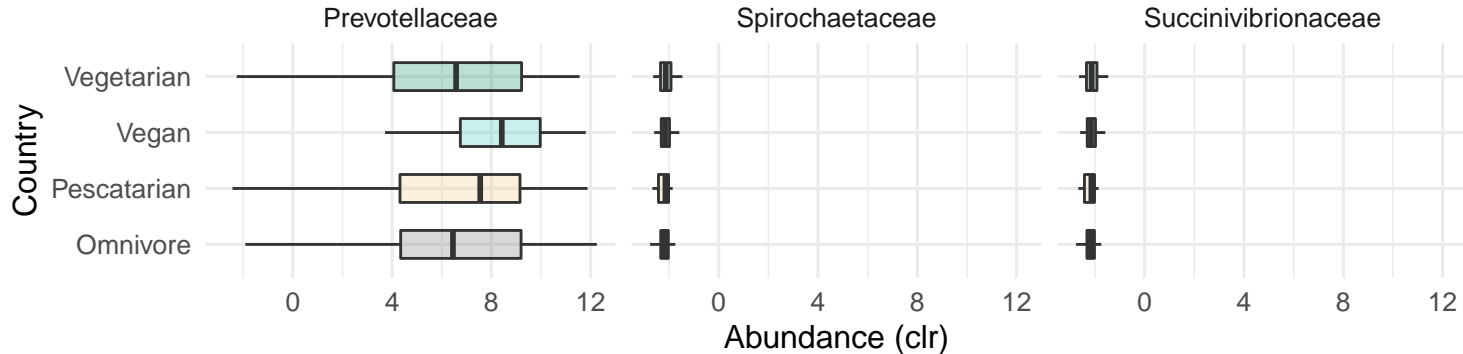

b

## BloSSUM taxa in NLD-VEGA

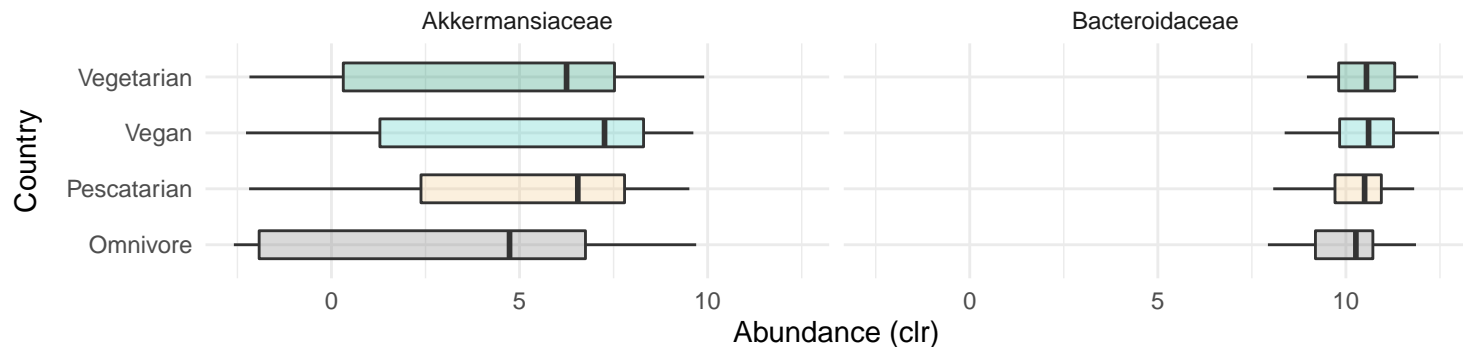

Supplement: FIG S2 [file msphere.00512-22-s0002.pdf]

Legend: Omnivore (pink), Pescatarian (orange), Vegan (light blue), Vegetarian (green)

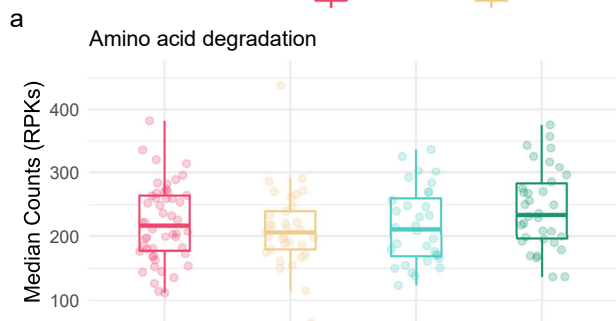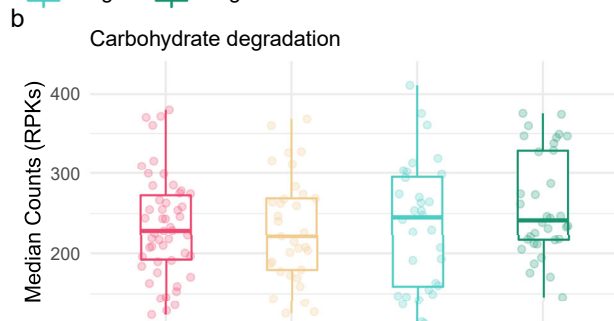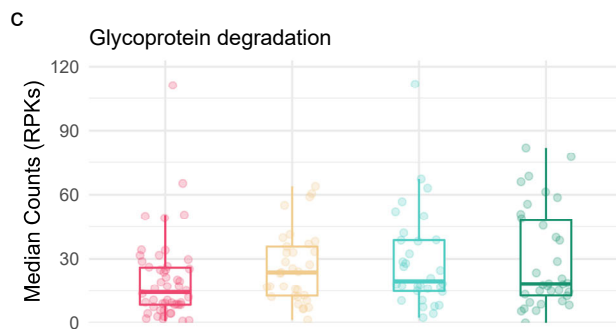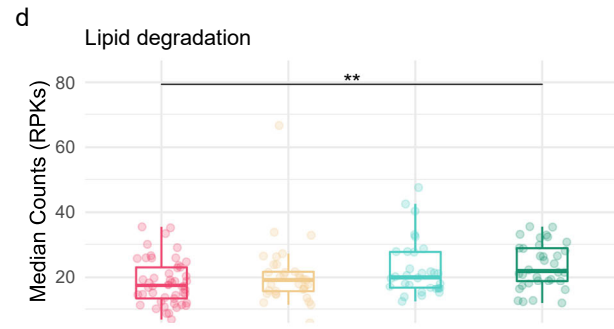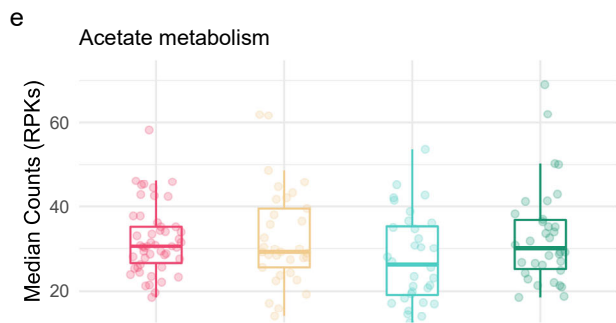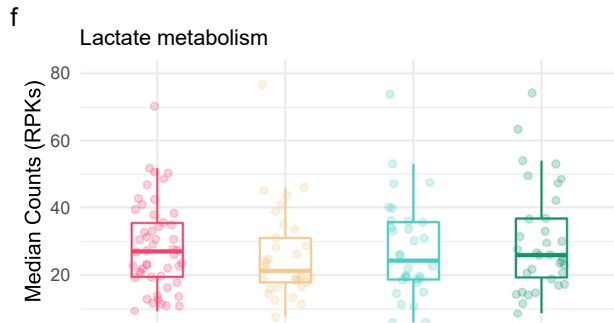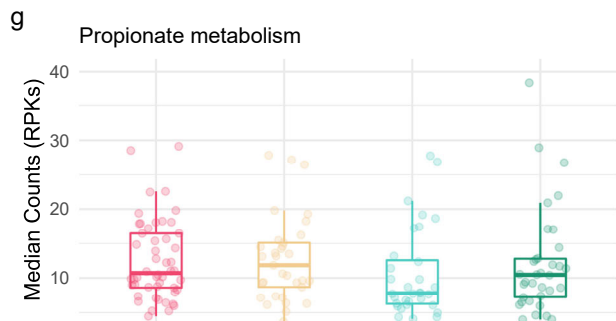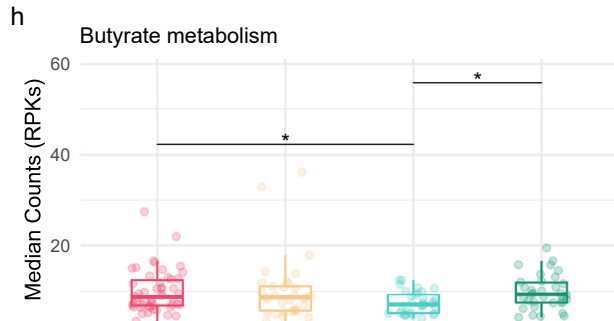

Supplement: FIG S3 [file msphere.00512-22-s0003.pdf]
