# Supplementary material for: Species-Specific Patterns of Gut Metabolic Modules in Dutch Individuals with Different Dietary Habits
Source: mSphere. 2022 Nov 17;7(6):e00512-22. doi: 10.1128/msphere.00512-22 (PMC9769759; doi:10.1128/msphere.00512-22)
Supplement: TABLE S1 [file msphere.00512-22-s0005.docx]

|  | Omnivore | Pescatarian | Vegetarian | Vegan |
| --- | --- | --- | --- | --- |
| Number of participants | 50 | 33 | 34 | 32 |
| Age in years, median (10th–90th percentile) | 47 (29–59) | 51 (29–62) | 45 (28–62) | 37 (29–56) |
| Male participants,(percentage) | 16 (32%) | 12 (36%) | 11 (32%) | 11 (34%) |
