# Supplementary material for: Species-Specific Patterns of Gut Metabolic Modules in Dutch Individuals with Different Dietary Habits
Source: mSphere. 2022 Nov 17;7(6):e00512-22. doi: 10.1128/msphere.00512-22 (PMC9769759; doi:10.1128/msphere.00512-22)
Supplement: TEXT S1 [file msphere.00512-22-s0004.docx]

# Supplementary methods

# Species-specific gut microbiome patterns in Dutch individuals with different dietary habits.

Sudarshan A. Shetty^1,2^, Paul B. Stege^3^, Joost Hordijk^1^, Esther Gijsbers^1^, Cindy M. Dierikx^1^, Engeline van Duijkeren^1^, Eelco Franz^1^, Rob J. L. Willems^3^, Fernanda L. Paganelli^3^, Susana Fuentes^1^*

1. Centre for Infectious Disease Control, National Institute for Public Health and the Environment (RIVM), the Netherlands.

2. Department of Medical Microbiology and Infection prevention, Virology and Immunology research Group, University Medical Center Groningen, the Netherlands.

3. Department of Medical Microbiology, UMC Utrecht, The Netherlands.

*Corresponding author: [susana.fuentes@rivm.nl](mailto:susana.fuentes@rivm.nl). National Institute for Public Health and the Environment (RIVM), Antonie van Leeuwenhoeklaan 9, 3721 MA Bilthoven, the Netherlands.

**Supplementary methods**

**Data used in this study**

Metagenomic raw data from a previously published study on 149 Dutch individuals from the general population (the “NLD-VEGA-study”) were used in this study (1, 2). Participants were categorized in four different diet groups: 1) omnivores (n=50), 2) pescatarians (n=33), 3) vegetarians (n=34) and 4) vegans (n=32). Details of the diet group classification and generation of metagenomic data are described previously (1) and Supplementary table S1.

For global comparison of gut microbiomes, we obtained taxonomic profiles from publicly available human gut metagenomic data by curatedMetagenomicData (v 2.99.1) (3). Details of the studies included in the analysis are provided in Supplementary table S1 (Refs. 7-22). Taxonomic profiles for these data were obtained using MetaPhlAn-3 (4). This dataset consisted of 2369 samples, of which 1940 were categorised as Westernized and 429 as non-Westernized. The age range was between 19 and 75 with a median of 44 years old. For some participants (n= 151; 6.4%) age information was not available. In both Westernized and non- Westernized groups, females were higher than males (59.8% and 52.6% respectively).

**Bioinformatics processing**

The quality of raw paired-end reads was investigated using fastqc. Raw reads were filtered and trimmed using BBTools BBDuk (https://jgi.doe.gov/data-and-tools/bbtools/bb-tools-user-guide/bbduk-guide/) module with following parameters minlen: 100; trimq: 18; maq: 20; ktrim: r; qtrim: rl; ftl: 0; ftr: 0; maxns: 0. Human DNA from data was removed with kneaddata using default parameters (4). Taxonomic composition and functional assignment were done using biobakery computational environment, MetaPhlAn-3 and HUMAnN-3 (4). The pathway profiles and gene family abundances were obtained from HUMAnN-3 (UniRef90) and species-linked gene families were linked to KEGG orthologs (KOs) using the *humann_regroup_table* function.

Following this, individual sample taxonomic and functional profiles were imported into R (v 4.0.5) for downstream analysis. The species-linked KO profile for each sample were used to calculated the median abundance of gut metabolic modules using omixrRpm (v0.3.2) (5). The gut metabolic module classification used in the analysis reported in this study is provided on the GitHub repository (<https://github.com/RIVM-IIV-Microbiome/VEGA-2021>).

**Data analysis**

Comparisons of community structure between samples was done using Aitchison distance to account for compositionality. Core microbiota analysis was done using the core() function in microbiome R package (v1.12.0) (6) with following cut-off, minimum relative abundance 0.0001% and 75% prevalence. For comparing similarity of taxonomic profiles of VEGA cohort with other samples in curatedMetagenomicData (v 2.99.1) (3), we used 1-Bray Curtis using relative abundance data. Pairwise comparisons were done using two-sided Wilcoxon test adjusted by the Benjamini & Hochberg method for multiple testing. Codes and scripts to reproduce the analysis are made available on GitHub (<https://github.com/RIVM-IIV-Microbiome/VEGA-2021>).

**Supplementary References**

1. Stege PB, Hordijk J, Shetty SA, Visser M, Viveen MC, Rogers MR, et al. Impact of long-term dietary habits on the human gut resistome in the Dutch population. Scientific reports. 2022;12(1):1-13.

2. Meijs AP, Gijsbers EF, Hengeveld PD, Veenman C, van Roon AM, van Hoek AH, et al. Do vegetarians less frequently carry ESBL/pAmpC-producing Escherichia coli/Klebsiella pneumoniae compared with non-vegetarians? Journal of Antimicrobial Chemotherapy. 2020;75(3):550-8.

3. Pasolli E, Schiffer L, Manghi P, Renson A, Obenchain V, Truong DT, et al. Accessible, curated metagenomic data through ExperimentHub. Nat Methods. 2017;14(11):1023.

4. Beghini F, McIver LJ, Blanco-Míguez A, Dubois L, Asnicar F, Maharjan S, et al. Integrating taxonomic, functional, and strain-level profiling of diverse microbial communities with bioBakery 3. Elife. 2021;10:e65088.

5. Darzi Y, Falony G, Vieira-Silva S, Raes J. Towards biome-specific analysis of meta-omics data. The ISME journal. 2016;10(5):1025.

6. Lahti L, Shetty SA. Introduction to the microbiome R package. Bioconductor2018.

7. De Filippis F, Pasolli E, Tett A, Tarallo S, Naccarati A, De Angelis M, et al. Distinct genetic and functional traits of human intestinal Prevotella copri strains are associated with different habitual diets. Cell host & microbe. 2019;25(3):444-53. e3.

8. Dhakan D, Maji A, Sharma AK, Saxena R, Pulikkan J, Grace T, et al. The unique composition of Indian gut microbiome, gene catalogue, and associated fecal metabolome deciphered using multi-omics approaches. Gigascience. 2019;8(3):giz004.

9. Hannigan GD, Duhaime MB, Ruffin MT, Koumpouras CC, Schloss PD. Diagnostic potential and interactive dynamics of the colorectal cancer virome. MBio. 2018;9(6):e02248-18.

10. Hansen L, Roager HM, Søndertoft NB, Gøbel RJ, Kristensen M, Vallès-Colomer M, et al. A low-gluten diet induces changes in the intestinal microbiome of healthy Danish adults. Nature communications. 2018;9(1):1-13.

11. Kaur K, Khatri I, Akhtar A, Subramanian S, Ramya T. Metagenomics analysis reveals features unique to Indian distal gut microbiota. PloS one. 2020;15(4):e0231197.

12. Keohane DM, Ghosh TS, Jeffery IB, Molloy MG, O’Toole PW, Shanahan F. Microbiome and health implications for ethnic minorities after enforced lifestyle changes. Nature Medicine. 2020;26(7):1089-95.

13. Zhernakova A, Kurilshikov A, Bonder MJ, Tigchelaar EF, Schirmer M, Vatanen T, et al. Population-based metagenomics analysis reveals markers for gut microbiome composition and diversity. Science. 2016;352(6285):565-9.

14. Liu W, Zhang J, Wu C, Cai S, Huang W, Chen J, et al. Unique features of ethnic Mongolian gut microbiome revealed by metagenomic analysis. Scientific reports. 2016;6(1):1-13.

15. Obregon-Tito AJ, Tito RY, Metcalf J, Sankaranarayanan K, Clemente JC, Ursell LK, et al. Subsistence strategies in traditional societies distinguish gut microbiomes. Nature communications. 2015;6:6505.

16. Pasolli E, Asnicar F, Manara S, Zolfo M, Karcher N, Armanini F, et al. Extensive unexplored human microbiome diversity revealed by over 150,000 genomes from metagenomes spanning age, geography, and lifestyle. Cell. 2019;176(3):649-62. e20.

17. Pehrsson EC, Tsukayama P, Patel S, Mejía-Bautista M, Sosa-Soto G, Navarrete KM, et al. Interconnected microbiomes and resistomes in low-income human habitats. Nature. 2016;533(7602):212-6.

18. Qin J, Li Y, Cai Z, Li S, Zhu J, Zhang F, et al. A metagenome-wide association study of gut microbiota in type 2 diabetes. Nature. 2012;490(7418):55-60.

19. Rosa BA, Supali T, Gankpala L, Djuardi Y, Sartono E, Zhou Y, et al. Differential human gut microbiome assemblages during soil-transmitted helminth infections in Indonesia and Liberia. Microbiome. 2018;6(1):1-19.

20. Rubel MA, Abbas A, Taylor LJ, Connell A, Tanes C, Bittinger K, et al. Lifestyle and the presence of helminths is associated with gut microbiome composition in Cameroonians. Genome biology. 2020;21(1):1-32.

21. Smits SA, Leach J, Sonnenburg ED, Gonzalez CG, Lichtman JS, Reid G, et al. Seasonal cycling in the gut microbiome of the Hadza hunter-gatherers of Tanzania. Science. 2017;357(6353):802-6.

22. Xie H, Guo R, Zhong H, Feng Q, Lan Z, Qin B, et al. Shotgun metagenomics of 250 adult twins reveals genetic and environmental impacts on the gut microbiome. Cell systems. 2016;3(6):572-84. e3.
