# Supplementary material for: Species-Specific Patterns of Gut Metabolic Modules in Dutch Individuals with Different Dietary Habits
Source: mSphere. 2022 Nov 17;7(6):e00512-22. doi: 10.1128/msphere.00512-22 (PMC9769759; doi:10.1128/msphere.00512-22)
Supplement: TABLE S2 [file msphere.00512-22-s0006.docx]

| Sr. No. | Study Name | Stool Samples used in this study | Country | PubmedID |
| --- | --- | --- | --- | --- |
| 1 | DeFilippisF_2019 | 97 | Italy | 30799264 (7) |
| 2 | DhakanDB_2019 | 87 | India | 30698687 (8) |
| 3 | HanniganGD_2017 | 25 | U.S.A. | 30459201 (9) |
| 4 | HansenLBS_2018 | 207 | Denmark | 30425247 (10) |
| 5 | KaurK_2020 | 30 | India | 32267865 (11) |
| 6 | KeohaneDM_2020 | 109 | Ireland | 32632193 (12) |
| 7 | LifeLinesDeep_2016 | 1039 | Netherlands | 27126040 (13) |
| 8 | LiuW_2016 | 110 | Mongolia | 27708392 (14) |
| 9 | Obregon-TitoAJ_2015 | 36 | Peru, U.S.A. | 25807110 (15) |
| 10 | PasolliE_2019 | 110 | Madagascar | 30661755 (16) |
| 11 | PehrssonE_2016 | 102 | Peru/El-Salvador | 27172044 (17) |
| 12 | QinJ_2012 | 186 | China | 23023125 (18) |
| 13 | RosaBA_2018 | 5 | Indonesia | 29486796 (19) |
| 14 | RubelMA_2020 | 68 | Cameroon | 32450885 (20) |
| 15 | SmitsSA_2017 | 32 | Tanzania | 28839072 (21) |
| 16 | XieH_2016 | 126 | Great Britain | 27818083 (22) |
